# Chest x-ray severity score in COVID-19 patients on emergency department admission: a two-centre study

**DOI:** 10.1186/s41747-020-00195-w

**Published:** 2020-12-15

**Authors:** Cristian Giuseppe Monaco, Federico Zaottini, Simone Schiaffino, Alessandro Villa, Gianmarco Della Pepa, Luca Alessandro Carbonaro, Laura Menicagli, Andrea Cozzi, Serena Carriero, Francesco Arpaia, Giovanni Di Leo, Davide Astengo, Ilan Rosenberg, Francesco Sardanelli

**Affiliations:** 1 Unit of Radiology, IRCCS Policlinico San Donato, Via Rodolfo Morandi 30, 20097 San Donato Milanese, Italy; 2 Unit of Radiology, Ospedale Lavagna, Via Don Giovanni Battista Bobbio 25, 16033 Lavagna, Italy; 3 Postgraduate School in Radiodiagnostics, Università degli Studi di Milano, Via Festa del Perdono 7, 20122 Milan, Italy; 4 Department of Biomedical Sciences for Health, Università degli Studi di Milano, Via Luigi Mangiagalli 31, 20133 Milan, Italy

**Keywords:** COVID-19, COVID-19 diagnostic testing, Diagnostic imaging, Chest x-ray, Severity score

## Abstract

**Background:**

Integration of imaging and clinical parameters could improve the stratification of COVID-19 patients on emergency department (ED) admission. We aimed to assess the extent of COVID-19 pulmonary abnormalities on chest x-ray (CXR) using a semiquantitative severity score, correlating it with clinical data and testing its interobserver agreement.

**Methods:**

From February 22 to April 8, 2020, 926 consecutive patients referring to ED of two institutions in Northern Italy for suspected SARS-CoV-2 infection were reviewed. Patients with reverse transcriptase-polymerase chain reaction positive for SARS-CoV-2 and CXR images on ED admission were included (295 patients, median age 69 years, 199 males). Five readers independently and blindly reviewed all CXRs, rating pulmonary parenchymal involvement using a 0–3 semiquantitative score in 1-point increments on 6 lung zones (range 0–18). Interobserver agreement was assessed with weighted Cohen’s *κ*, correlations between median CXR score and clinical data with Spearman’s *ρ*, and the Mann-Whitney *U* test.

**Results:**

Median score showed negative correlation with SpO_2_ (*ρ* = -0.242, *p* < 0.001), positive correlation with white cell count (*ρ* = 0.277, *p* < 0.001), lactate dehydrogenase (*ρ* = 0.308, *p* < 0.001), and C-reactive protein (*ρ* = 0.367, *p* < 0.001), being significantly higher in subsequently dead patients (*p* = 0.003). Considering overall scores, readers’ pairings yielded moderate (*κ* = 0.449, *p* < 0.001) to almost perfect interobserver agreement (*κ* = 0.872, *p* < 0.001), with better interobserver agreement between readers of centre 2 (up to *κ* = 0.872, *p* < 0.001) than centre 1 (*κ* = 0.764, *p* < 0.001).

**Conclusions:**

Proposed CXR pulmonary severity score in COVID-19 showed moderate to almost perfect interobserver agreement and significant but weak correlations with clinical parameters, potentially furthering CXR integration in patients’ stratification.

## Key points

- Chest x-ray is a first-choice imaging modality for the evaluation of COVID-19 pneumonia.
- Proposed semiquantitative chest x-ray severity score showed weak but significant correlations with clinical parameters.
- Chest x-ray severity score of pulmonary COVID-19 involvement showed substantial interobserver agreement.

## Background

In December 2019, a new beta coronavirus causing severe acute respiratory syndrome (SARS-CoV-2) was identified as the causative agent of coronavirus disease 2019 (COVID-19) [1], becoming a pandemic since March 11, 2020, as announced by the World Health Organization [2].

Reverse transcriptase-polymerase chain reaction (RT-PCR)—even though burdened by sensitivity limitations [3, 4]—is considered the reference standard to diagnose SARS-CoV-2 infection, while the diagnostic role of chest imaging—including chest x-ray (CXR) and computed tomography—is debated [5–16]. Of note, CXR is the first-choice imaging modality for evaluating acute respiratory illness and can play a role in the follow-up during and after treatment [5, 8, 11–13, 17–25].

Accurate stratification of COVID-19 patients by severity of their conditions is paramount to assure correct allocation of resources [26]. In particular, one of the first parameters investigated for each patient on admission is the value of peripheral oxygen saturation (SpO_2_), which frequently mirrors the degree of lung function impairment. Along with concurrent comorbidities, SpO_2_ largely determines the need of a COVID-19 patient to be transferred to intensive care units [1, 27]. In this view, there is a need to early stratify pulmonary involvement in COVID-19 patients: attaining this objective with CXR could add to the already established diagnostic relevance of this technique a role—shared with other clinical parameters commonly acquired on emergency department (ED) admission—in stratifying patients according to disease severity [17–25], potentially further curtailing the use of CT and the related workflow burden.

The aim of this study was therefore to assess the extent of pulmonary abnormalities in COVID-19 patients applying a semiquantitative severity score on CXRs performed on ED admission, testing its interobserver agreement and its correlation with clinical data obtained on ED admission.

## Methods

This Ethics Committee-approved retrospective observational study includes two different institutions from Northern Italy, IRCCS Policlinico San Donato (San Donato Milanese, Italy), centre 1, and Ospedale di Lavagna (Lavagna, Italy), centre 2. During the COVID-19 pandemic peak, centre 1 has been a COVID-19-dedicated hospital, less than 25 mi from the first Italian hotspot of Codogno, while centre 2 has been a non-dedicated hospital, in a region near Lombardy, almost 100 mi away from Milan.

### Study population

We retrospectively reviewed clinical and imaging records of all patients referring to the ED of the two institutions for suspected SARS-CoV-2 infection between February 22 and April 8, 2020. Each patient underwent a pharyngeal swab for RT-PCR and a bedside CXR within a maximum time interval of 12 h. CXRs were used in both centres to address known shortcomings of RT-PCR diagnostic performance and limitations in its turnaround time. CXRs were performed at bedside in the ED isolation rooms of each centre, using one of two different systems at centre 1 (Digital GM85, Samsung Healthcare, Seoul, South Korea; Digital FDR Go PLUS, Fujifilm, Tokyo, Japan), and one system at centre 2 (Easyslide 30, SMAM, Monza, Italy). Only patients with subsequent RT-PCR-confirmed SARS-CoV-2 infection were included in our study.

Demographic and clinical data were retrieved from the electronic system of each centre, including blood oxygen saturation (SpO_2_) and body temperature on ED admission, comorbidities, and arterial and venous blood tests.

### Chest x-ray review

**Fig. 1:**
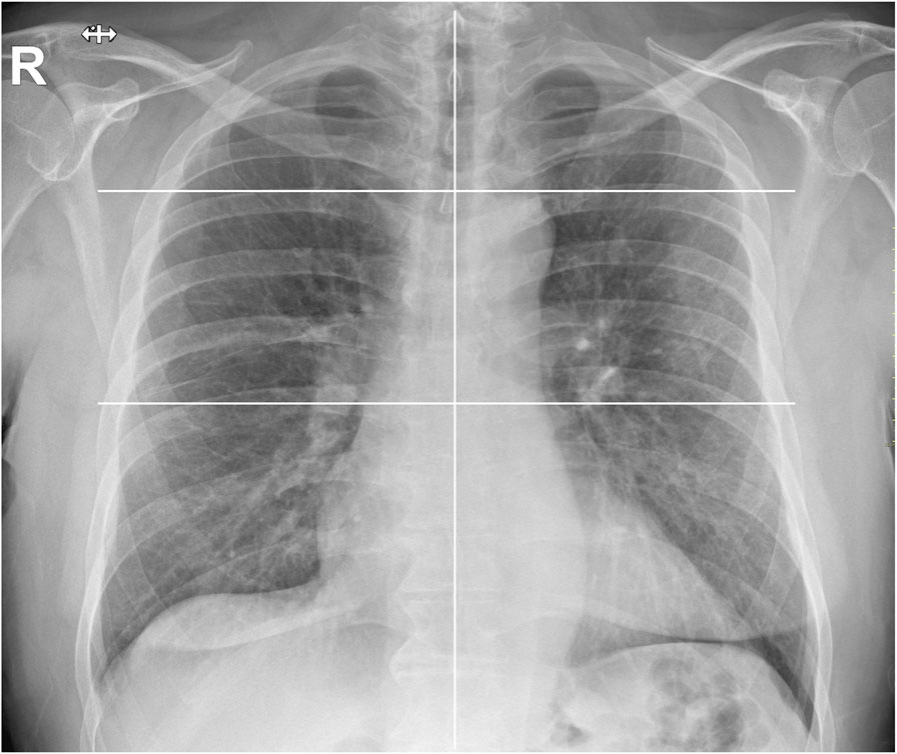
CXR subdivision, with three parts for each lung: superior zone (from the lung apex to the aortic arch profile), middle zone (lung hilum, from the aortic arch profile to the inferior margin of the left pulmonary hilum), and inferior zone (from the inferior margin of the left pulmonary hilum to the diaphragm)

**Fig. 2:**
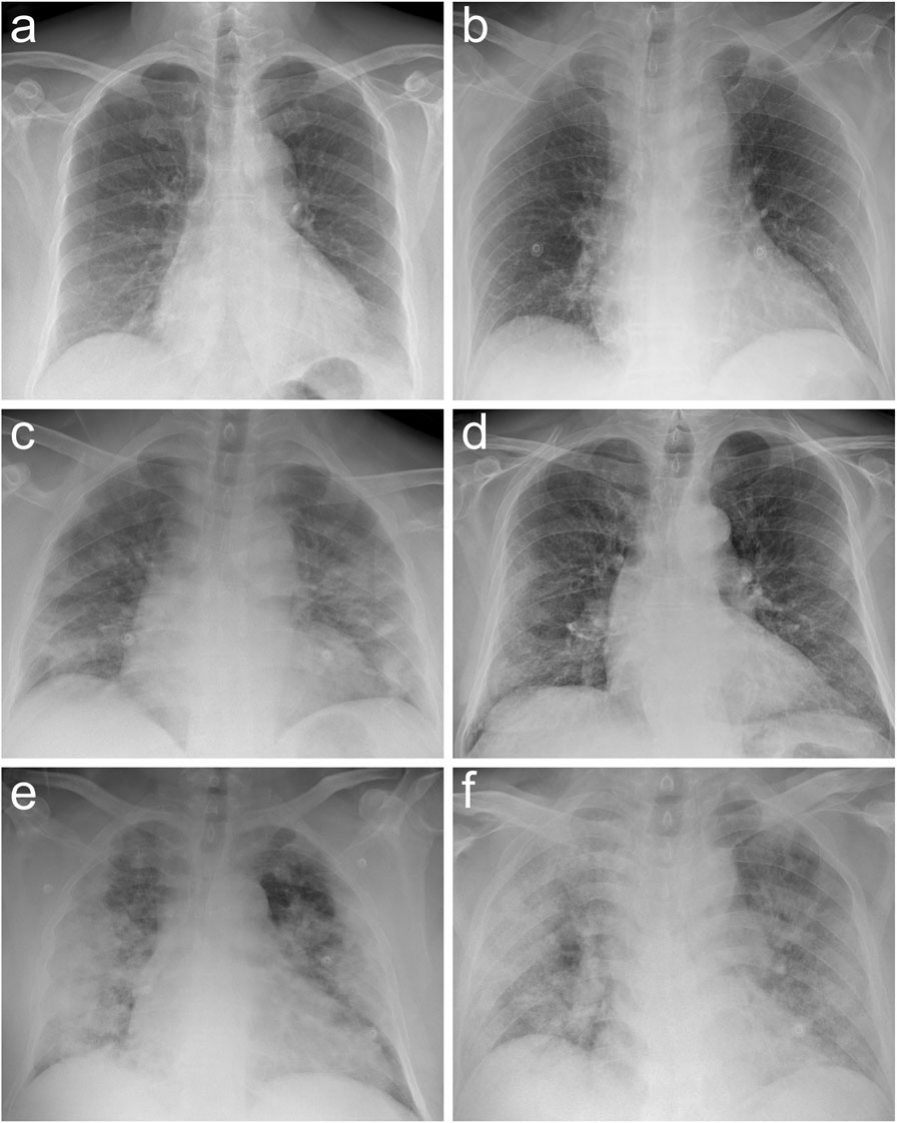
CXR scoring in three COVID-19 patients with different degrees of lung parenchymal involvement. The CXR score for each patient was (upper zones R-L; middle zones R-L; lower zones R-L): **a** 0-0, 0-0, 1-1 (total 2); **b** 0-0, 2-2, 1-1 (total 6); **c** 1-1, 2-3, 2-3 (total 12); **d** 1-1, 2-2, 2-2 (total 10); **e** 2-2, 3-3, 3-3 (total 16); **f** 3-1, 3-3, 3-3 (total 17)

Five readers, two radiologists from centre 1 (C.M. and L.M., with 6 and 13 years of experience in chest imaging, respectively) and three radiologists from centre 2 (D.A., A.V., and F.Z., with 10, 15, and 5 years of experience in chest imaging, respectively) independently and blindly reviewed all anonymised and randomised CXRs from the two centres. The readers rated pulmonary parenchymal involvement using a semiquantitative severity score, subdividing each lung into three zones (Fig. 1): upper zone (from the lung apex to the aortic arch profile), middle zone (from the aortic arch profile to the lower margin of the left pulmonary hilum), and lower zone (from the lower margin of the left pulmonary hilum to the diaphragm). For each zone, a score on a scale from zero to three in 1-point increments was assigned: 0, normal lung parenchyma; 1, interstitial involvement only; 2, presence of radiopacity for less than 50% of the visible lung parenchyma; 3, presence of radiopacity for 50% or more than 50% of the visible lung parenchyma (Fig. 2).

### Statistical analysis

Data were reported as median and interquartile range (IQR), with calculation of the lower and upper 95% confidence interval (CI) when appropriate. Correlations between overall median CXR severity score and clinical data were assessed using the Spearman’s rank order correlation and the Mann-Whitney *U* test. Considering the semiquantitative rather than ordinal nature of our score, particularly in its overall formulation, intraclass correlation coefficients with a quadratic-weighted Cohen’s *κ* statistics were used to assess interobserver agreement, *κ* values being interpreted according to the Landis and Koch scale [28]. Statistical analyses were performed using the SPSS v.26.0 software (IBM SPSS Inc., Chicago, IL, USA). Statistical significance was set at *p* values < 0.05.

## Results

During the study period, a total of 926 patients (676 at centre 1, 250 at centre 2) presented at the ED of the two centres. We ultimately included in this study 295 of them (201 from centre 1 and 94 from centre 2) having a SARS-CoV-2 diagnosis confirmed by RT-PCR and available CXR images. Of these 295 patients (199 males, median age 69 years, interquartile range [IQR] 56–79 years), the 201 patients from centre 1 were 140 males and 61 females (median age 65 years, IQR 58–78), while the 94 patients from centre 2 were 59 males and 35 females (median age 68, IQR 52–80).

On ED admission, median SpO_2_ value for all 295 patients was 93% (IQR 89.2–96%) and median body temperature was 37.7 °C (IQR 37.0–38.2 °C). Data on comorbidities and symptoms were available for centre 1 only, due to lack of electronic medical records at centre 2, while clinical and laboratory data were available for all 295 patients (Table 1). At centre 1, at least one comorbidity was found in 116 out of 201 patients (58%) with a median 1 comorbidity per-patient (IQR 0–2), hypertension being the most frequent (86/201 patients, 43%), followed by cardiovascular disease (41/201 patients, 20%), previous malignancy history (11 patients, 6%), and chronic obstructive pulmonary disease (5 patients, 3%). On ED admission at centre 1, the most common symptoms were fever (184/201 patients, 92%), cough (128/201 patients, 63%), and dyspnea (126/201 patients, 63%), variously combined with other symptoms as shown in Table 2. Median hospitalisation length was 18 days (IQR 12–24 days).

**Table 1:** Laboratory and clinical characteristics on admission of the 295 patients included in the study

	Median value	Interquartile range
SpO_2_ (%)	93.0	89.2–96.0
Leucocytes (10^3^/μL)	6.5	4.8–8.7
Lymphocytes (10^3^/μL)	1.1	0.8–2.4
Platelets (10^3^/μL)	186	142–254
Lactate dehydrogenase (U/L)	489	363–704
C-reactive protein (mg/dL)	9.0	4.4–15.5
pH	7.471	7.436–7.498
pCO_2_ (mmHg)	33.5	29.8–37.1
pO_2_ (mmHg)	63.8	55.4–73.5
HCO_3_^−^ (mmol/L)	24.8	22.2–26.5
Lactates (mmol/L)	1.32	0.91–1.80

**Table 2:** Symptoms on admission of the 201 patients from centre 1

Patients	Symptoms	Fever	Cough	Dyspnea	Asthenia	Diarrhea	Vomit	MSK pain	Syncope
77 (38.3%)	3	X	X	X					
39 (19.4%)	2	X	X						
29 (14.4%)	2	X		X					
21 (10.4%)	1	X							
6 (3.0%)	1			X					
3 (1.5%)	3	X		X	X				
3 (1.5%)	2		X	X					
3 (1.5%)	2	X			X				
2 (1.0%)	4	X	X	X		X			
2 (1.0%)	3	X	X				X		
2 (1.0%)	3	X		X				X	
2 (1.0%)	1				X				
2 (1.0%)	1		X						
1 (0.5%)	3			X	X	X			
1 (0.5%)	3	X	X		X				
1 (0.5%)	3	X	X			X			
1 (0.5%)	3	X				X	X		
1 (0.5%)	3	X		X		X			
1 (0.5%)	3	X		X					X
1 (0.5%)	2		X			X			
1 (0.5%)	2			X	X				
1 (0.5%)	2	X					X		
1 (0.5%)	1					X			
Total patients		**184** (91.5%)	**128** (63.7%)	**126** (62.7%)	**11** (5.5%)	**8** (4.0%)	**4** (2.0%)	**2** (1.0%)	**1** (0.5%)

*MSK* musculoskeletal

As of June 30, 2020, after a median follow-up length of 104 days (IQR 100–109 days), censoring was applied, recording 58 deaths during hospitalisation; these patients had a significantly higher median CXR severity score on admission (16.5, IQR 13–20) than surviving patients (15, IQR 13–16, *p* = 0.003), being also significantly older (*p* < 0.001) than surviving patients (median age 76 years with IQR 70–83 years, and median age 66 years with IQR 55–75 years, respectively).

Overall, median CXR severity score was 8 (IQR 6–11), without any significant difference between men and women (*p* = 0.758), showing however a significant but weak correlation with age (*ρ* = 0.177, *p* = 0.002).

### Correlation between CXR severity score and clinical data

Median CXR severity score showed weak correlations with clinical data, in particular significant negative correlations with SpO_2_ on ED admission (*ρ* = -0.242, *p* < 0.001), lymphocytes (*ρ* = -0.162, *p* = 0.005), and PaO_2_ at blood gas analysis (*ρ* = -0.203, *p* = 0.004), significant positive correlations with total white blood cell count (*ρ* = 0.277, *p* < 0.001), platelets (*ρ* = 0.161, *p* = 0.006), lactate dehydrogenase (*ρ* = 0.308, *p* < 0.001), and C-reactive protein values (*ρ* = 0.367, *p* < 0.001). Among other arterial blood gas values on ED admission, none except lactate levels (*ρ* = 0.257, *p* < 0.001) showed a significant correlation with CXR severity score: pH (*ρ* = 0.129, *p* = 0.060), pCO_2_ (*ρ* = 0.031, *p* = 0.657), HCO_3_^−^ (*ρ* = 0.028, *p* = 0.682).

### Interobserver agreement

Considering the overall severity score for all lung zones, interobserver agreement between the five readers ranged from moderate (*κ* = 0.449, *p* < 0.001, comparing reader 1 from centre 1 and reader 3 from centre 2) to almost perfect (*κ* = 0.872, *p* < 0.001, comparing reader 2 and reader 3 from centre 2) with a strong overall intraclass correlation coefficient (0.639, IQR 0.417–0.769 with *p* < 0.001).

Considering interobserver agreement between readers from the same institution, the two radiologists from centre 1 showed substantial interobserver agreement (*κ* = 0.764, *p* < 0.001) and the three radiologists from centre 2 ranged from substantial interobserver agreement (reader 1 *versus* reader 3, *κ* = 0.792, *p* < 0.001) to almost perfect interobserver agreement (reader 2 *versus* reader 3, *κ* = 0.872, *p* < 0.001). Table 3 shows all quadratic-weighted *κ* values for each pair of readers.

**Table 3:** Quadratic-weighted Cohen’s *κ* values of interobserver agreement for each pair of the five readers

	**Centre 1 reader 1**			
**Centre 1 reader 1**	-	**Centre 1 reader 2**		
**Centre 1 reader 2**	0.764 (0.712–0.816)	**-**	**Centre 2 reader 1**	
**Centre 2 reader 1**	0.528 (0.473–0.582)	0.618 (0.554–0.682)	**-**	**Centre 2 reader 2**
**Centre 2 reader 2**	0.478 (0.421–0.535)	0.615 (0.546–0.684)	0.834 (0.800–0.869)	**-**
**Centre 2 reader 3**	0.449 (0.387–0.511)	0.579 (0.503–0.656)	0.792 (0.739–0.844)	0.872 (0.834–0.910)

Values in parentheses represent the lower and upper 95% confidence interval

Considering interobserver agreement for each lung zone between the five readers, readers from centre 2 had higher intraclass correlation coefficients compared to centre 1, both overall and for each zone, with higher overall intraclass correlation coefficients for the evaluation of middle lung zones compared to upper and lower ones (Table 4).

**Table 4:** Intraclass correlation coefficients in the overall and zone-specific lung evaluation

	Intraclass correlation coefficients					
	Overall	***p***	Centre 1	***p***	Centre 2	***p***
All lung zones	0.639 (0.417–0.769)	< 0.001	0.757 (0.611–0.840)	< 0.001	0.783 (0.736–0.825)	< 0.001
Upper right zone	0.564 (0.421–0.671)	< 0.001	0.604 (0.505–0.686)	< 0.001	0.656 (0.590–0.717)	< 0.001
Middle right zone	0.607 (0.483–0.701)	< 0.001	0.573 (0.426–0.682)	< 0.001	0.697 (0.636–0.752)	< 0.001
Lower right zone	0.512 (0.360–0.629)	< 0.001	0.400 (0.006–0.640)	< 0.001	0.613 (0.542–0.679)	< 0.001
Upper left zone	0.475 (0.327–0.595)	< 0.001	0.532 (0.377–0.649)	< 0.001	0.558 (0.481–0.631)	< 0.001
Middle left zone	0.651 (0.570–0.718)	< 0.001	0.700 (0.621–0.765)	< 0.001	0.705 (0.633–0.751)	< 0.001
Lower left zone	0.574 (0.473–0.658)	< 0.001	0.538 (0.121–0.743)	< 0.001	0.636 (0.568–0.700)	< 0.001

Values in parentheses represent the lower and upper 95% confidence interval

## Discussion

COVID-19 infection has frequently represented a scarcely manageable challenge for healthcare systems, in particular for EDs and intensive care units [26]. In this scenario, it is paramount to identify the most cost-effective procedures to be included in ED workflow and, at the same time, to reduce as much as possible the contact between healthcare workers and patients and between patients themselves [3, 29–31].

Literature on COVID-19 imaging has been chiefly focused on CT [5, 10, 15]. Only a comparatively lower number of studies have investigated the role of CXR, even if CXR is usually the first examination for patients entering ED for suspected SARS-CoV-2 infection, being also characterised by simpler logistics and usage [3, 5, 6, 22, 31].

Moreover, the high sensitivity of CT is counterbalanced by a lower specificity [15], and its routine use is jeopardised by logistic difficulties brought about by the need of different pathways for COVID-19 patients to avoid secondary patient and staff exposure, by the need of providing a number of undeferrable CT examinations for non-COVID-19 patients, by complex and time-consuming room and unit sanitisation procedures, and by CT scanners relatively lower availability. In such setting, CXR, especially if performed with portable radiological equipment, could better match smooth workflow requirements.

Since the number of COVID-19-related hospitalisation has constantly increased in the past few months, there is also an urgent need to improve risk stratification, fostering a more specifically tailored patient management [17, 24, 26]. An important point to ensure rapid stratification would be to assess the potential integration of CXR results (*i.e.*, the stratification of pulmonary parenchymal involvement) with clinical data routinely obtained on ED admission. In particular, we chose to address the issue of interobserver agreement evaluation of pulmonary parenchymal involvement between more than two readers and outside expert readers. This was done to mirror CXR interpretation conditions that were (at least in Italy) frequently observed during the first pandemic peak, when radiologists of wide-ranging experience on CXR interpretation were tasked to report CXRs of suspected or confirmed COVID-19 patients, even if their previous day-to-day clinical activity was not focused on chest imaging. Always considering the need to contextualise our score in an ED setting, we focused our research on quickly and easily obtainable laboratory parameters rather than on anamnestic information, far more difficult to retrieve in a pandemic scenario with high inflow of patients to the ED. These laboratory parameters were chosen among those best representing the baseline clinical situation of a COVID-19 patient and those having an established and close-knit interplay with CXR findings in the first-line ED evaluation of patients with acute respiratory illness.

The integration of CXR with these parameters can only be attained with a standardisation of the interpretation of imaging findings, making them “ready to match” with clinical parameters. We therefore devised a scoring system that would be easy to adopt, reproducible, and representative of the severity of lung parenchyma involvement. Distribution of lesions in our study confirmed the pattern already described in recent literature, with higher involvement of lower lung areas and only few patients presenting pleural effusion [23, 24].

Our proposed severity score was found to significantly but weakly correlate with the main clinical parameters routinely considered to differentiate patients who need hospitalisation and patients that could be treated at home, such as SpO_2_ (even though the significance in that case is only borderline), white blood cell count, and C-reactive protein. The weak nature of these correlations could be explained first by considering that a large number of pre-existing factors and frailties such as comorbidities, weight, muscle mass, and age, strongly interplay between pneumonia extent and clinical and laboratory parameters of patients with COVID-19 needing hospitalisation [32]. Moreover, the increasingly demonstrated impact of pulmonary arterial thrombosis, which has shown little to none correlation with pneumonia extent [33] and can occur in lung parenchymal areas unaffected by pneumonia [34–37], represents a sizeable contribution to the mismatch between clinical parameters and pneumonia extent.

The two-centre multi-reader design of our study explains the overall substantial interobserver agreement, ranging from moderate (*κ* = 0.449) to almost perfect (*κ* = 0.872), with better results between readers of the same centre. The intraclass correlation coefficient observed for zone-specific scores was generally better for middle lung zones: this could be explained considering that upper and lower zones more frequently present findings interpreted as atelectasis lines or fibrotic thickening, rated with wider range of severity score (Table 4). We should also consider that, this being a novel severity score, a better interobserver agreement could be reached after more practice.

This study has limitations. First is its retrospective design and the limited availability of anamnestic information for one of the two centres. Second is the x-ray equipment difference between the two centres, possibly limiting the reproducibility of CXR findings. Third, the choice of including only SARS-CoV-2 positive (and subsequently hospitalised) patients in our study could have hindered a higher reproducibility of our score, being negative CXRs theoretically easier to recognise and score.

In conclusion, our proposed CXR severity score of pulmonary COVID-19 involvement showed moderate to almost perfect interobserver agreement and allowed to stratify disease extent, showing significant but weak correlations with clinical parameters. Potential extension of the role of CXR in patient management should be explored in larger studies.

## Data Availability

All data generated, obtained, or analysed during this study are included in this published article.

## Abbreviations

COVID-19: Coronavirus disease 2019
CXR: Chest x-ray
ED: Emergency department
IQR: Interquartile range
MSK: Musculoskeletal
RT-PCR: Reverse transcriptase-polymerase chain reaction
SARS-CoV-2: Severe acute respiratory syndrome
SpO_2_: Peripheral oxygen saturation
